# Artificial intelligence-supported lung cancer detection by multi-institutional readers with multi-vendor chest radiographs: a retrospective clinical validation study

**DOI:** 10.1186/s12885-021-08847-9

**Published:** 2021-10-18

**Authors:** Daiju Ueda, Akira Yamamoto, Akitoshi Shimazaki, Shannon Leigh Walston, Toshimasa Matsumoto, Nobuhiro Izumi, Takuma Tsukioka, Hiroaki Komatsu, Hidetoshi Inoue, Daijiro Kabata, Noritoshi Nishiyama, Yukio Miki

**Affiliations:** 1 Department of Diagnostic and Interventional Radiology, Graduate School of Medicine, Osaka City University, Osaka, Japan; 2 Department of Surgery, Graduate School of Medicine, Osaka City University, Osaka, Japan; 3 Department of Medical Statistics, Graduate School of Medicine, Osaka City University, Osaka, Japan

**Keywords:** Model validation, Chest radiography, Lung Cancer, Artificial intelligence, Deep learning, Computer-assisted detection

## Abstract

**Background:**

We investigated the performance improvement of physicians with varying levels of chest radiology experience when using a commercially available artificial intelligence (AI)-based computer-assisted detection (CAD) software to detect lung cancer nodules on chest radiographs from multiple vendors.

**Methods:**

Chest radiographs and their corresponding chest CT were retrospectively collected from one institution between July 2017 and June 2018. Two author radiologists annotated pathologically proven lung cancer nodules on the chest radiographs while referencing CT. Eighteen readers (nine general physicians and nine radiologists) from nine institutions interpreted the chest radiographs. The readers interpreted the radiographs alone and then reinterpreted them referencing the CAD output. Suspected nodules were enclosed with a bounding box. These bounding boxes were judged correct if there was significant overlap with the ground truth, specifically, if the intersection over union was 0.3 or higher. The sensitivity, specificity, accuracy, PPV, and NPV of the readers’ assessments were calculated.

**Results:**

In total, 312 chest radiographs were collected as a test dataset, including 59 malignant images (59 nodules of lung cancer) and 253 normal images. The model provided a modest boost to the reader’s sensitivity, particularly helping general physicians. The performance of general physicians was improved from 0.47 to 0.60 for sensitivity, from 0.96 to 0.97 for specificity, from 0.87 to 0.90 for accuracy, from 0.75 to 0.82 for PPV, and from 0.89 to 0.91 for NPV while the performance of radiologists was improved from 0.51 to 0.60 for sensitivity, from 0.96 to 0.96 for specificity, from 0.87 to 0.90 for accuracy, from 0.76 to 0.80 for PPV, and from 0.89 to 0.91 for NPV. The overall increase in the ratios of sensitivity, specificity, accuracy, PPV, and NPV were 1.22 (1.14–1.30), 1.00 (1.00–1.01), 1.03 (1.02–1.04), 1.07 (1.03–1.11), and 1.02 (1.01–1.03) by using the CAD, respectively.

**Conclusion:**

The AI-based CAD was able to improve the ability of physicians to detect nodules of lung cancer in chest radiographs. The use of a CAD model can indicate regions physicians may have overlooked during their initial assessment.

**Supplementary Information:**

The online version contains supplementary material available at 10.1186/s12885-021-08847-9.

## Background

Chest radiography is one of the most basic imaging tests in medicine and is the most common examination in routine clinical work such as screening for chest disease, diagnostic workup, and observation. One of the features physicians look for in these chest radiographs is nodules—an indicator of lung cancer, which has the highest mortality rate in the world [1]. In practice, low-dose CT is recommended [2] for lung cancer screening for at-risk individuals rather than chest radiography despite a false-positive rate of approximately 27% [2, 3]. Several studies concluded that low-dose CT was superior to radiographs which had a sensitivity of 36–84% [4–7], varying widely according to tumour size, study population, and reader performance. Other studies showed that 19–26% of lung cancers visible on chest radiographs were actually missed at the time of initial reading [6, 8]. However, chest radiography remains the primary diagnostic imaging test for chest conditions because of its advantages over chest CT, including ease of access, lower cost, and lower radiation exposure. Notably, the higher number of chest radiographs per capita than chest CT indicates that chest radiography has more opportunities to detect lung abnormalities in individuals who are not considered at risk, leading to a diagnostic chest CT.

Since the first computer-assisted detection (CAD) technique for chest radiography was reported in 1988 [9], there have been various developments designed to improve physicians’ performance [10–14]. Recently, the application of deep learning (DL), a field of artificial intelligence (AI) [13, 15], has led to dramatic, state-of-the-art improvements in visual object recognition and detection. Automated feature extraction, a critical component of DL, has great potential for application in the medical field [16], especially in radiology [17]. CADs using DL have routinely surpassed the performance of traditional methods. There were two studies which showed that a DL-based CAD may increase physicians’ sensitivity for lung cancer detection from chest radiography [18, 19]. However, these studies only compared the performance of radiologists. The American College of Radiology recommends that radiologists report on all diagnostic imaging [20], but there is a significant shortage of radiologists [21, 22]. In their absence, general physicians must interpret radiographs themselves. Patient safety can be improved either by improving the diagnostic accuracy of these physicians or by implementing systems that ensure that initial misinterpretations are corrected before they adversely affect patient care [23]. There are multiple causes of error in interpretating radiographs, but the most common one is recognition error. In other words, it refers to the inability to recognize an anomaly. Moreover, lung cancer was cited as the sixth most common cause for medicolegal action against physicians. The majority of the actions regarding missed lung cancer involved chest radiographs (90%) [24]. Thus, reading chest radiographs is important for general physicians, however there were no studies evaluating if an AI-based CAD could support not only radiologists, but also general physicians.

The purpose of the present study was to validate a commercially available AI-based CAD that achieved higher performance in detecting lung cancer from chest radiographs. To investigate the ability of this CAD as a support tool, we conducted a multi-vendor, retrospective reader performance test comparing both radiologist and general physicians’ performance before and after using the CAD.

## Methods

### Study design

A multi-vendor, retrospective clinical validation study comparing the performance of physicians before and after using the CAD was conducted to evaluate the capability of the CAD to assist physicians in detecting lung cancers on chest radiographs. Readers of varying experience level and specialization were included to determine if use of this model on regularly collected radiographs could benefit general physicians. This CAD is commercially available in Japan. The Osaka City University Ethics Board reviewed and approved the protocol of the present study. Since the chest radiographs used in the study had been acquired during daily clinical practice, the need for informed consent was waived by the ethics board. We have created this article in compliance with the STARD checklist [25].

### Datasets

To evaluate the AI-based CAD, chest radiographs of posterior-anterior view were retrospectively collected. Chest radiographs with lung cancers were consecutively collected from patients who had been subsequently surgically diagnosed with lung cancer between July 2017 and June 2018 at Osaka City University Hospital, which provides secondary care. The corresponding chest CT, taken within 14 days of the radiograph, were also collected. Chest radiographs with no findings were consecutively collected from patients who reported no nodule/mass finding by chest CT taken within 14 days at the same hospital. Detailed criteria are shown in Additional_File_1. Since the study included patients who visited our institution for the first time, there was no patient overlap among the datasets. Radiographs were taken using a DR CALNEO C 1417 Wireless SQ (Fujifilm Medical), DR AeroDR1717 (Konica Minolta), or DigitalDiagnost VR (Philips Medical Systems).

### Eligibility criteria and ground truth labelling

The eligibility criteria for the radiographs were as follows: (1) Mass lesions larger than 30 mm in size were excluded. (2) Metastatic lung cancer that was not primary to the lung was excluded. (3) Lung cancers showing anything other than nodular lesions on radiograph were excluded. (4) Nodules in the chest radiographs were annotated with bounding box, referring to chest CT images by two board-certificated radiologists, who had six years (D.U.) and five years (A.S.) of experience interpreting chest radiographs. Ground glass nodules with a diameter of less than 5 mm were excluded even if they were visible on CT, as they are not considered visible on chest radiographs. When there was disagreement between the annotating radiologists, consensus was achieved by discussion. Chest radiographs with lung cancer presenting nodules, their bounding boxes, and normal chest radiographs were combined to form a test dataset.

### The artificial intelligence-based computer-assisted detection model

The AI-based CAD used in this study is EIRL Chest X-ray Lung nodule (LPIXEL Inc.), commercially available in Japan as of August 2020 as a screening device to find primary lung cancer. The CAD was developed based on an encoder-decoder network categorizing segmentation technique in DL. The CAD was configured to display bounding boxes on all areas of suspected cancer in a radiograph. In the process of internal CAD, the areas suspected of being cancer on chest radiograph were segmented, and the maximum horizontal and vertical diameters of the segmented area are displayed as a bounding box.

### Reader performance test

To evaluate the capability of the CAD to assist physicians, a reader performance test comparing physician performance before and after use of the CAD was conducted. This CAD is certified as a medical software for use by physicians as a second opinion. In other words, physicians first read a chest radiograph without CAD, and then check the CAD output to make a final diagnosis. A total of eighteen readers (nine general physicians and nine radiologists from nine medical institutions) each interpreted the test dataset. The readers had not previously interpreted the same radiographs, did not know the ratio of malignant to normal cases, and clinical information regarding the radiographs was not made available to them. This process was double blinded for the examiners and the reading physicians.

The study protocol was as follows: (1) Each reader was individually trained with 30 radiographs outside the test dataset to familiarize them with the evaluation criteria and use of the CAD. (2) The readers interpreted the radiographs without using the AI-based CAD. If the reader concluded that there was a nodule in the image, then the lesion was annotated with a bounding box on the radiograph. Because the model was designed to produce bounding boxes on all areas that are considered to be positive, we instructed the readers to provide as many bounding boxes as they deemed necessary. (3) The CAD was then applied to the radiograph. (4) The reader interpreted the radiograph again, referring to the output of the CAD. If the reader changed their opinion, he or she annotated again or deleted the previous annotation. (5) The boxes annotated by the reader before and after use of the AI-based CAD were judged correct if the overlap, measured by the intersection over union (IoU), was 0.3 or higher. This value was chosen to meet a stricter standard based on the results from previous studies (Supplementary methods in Additional_File_1).

### Statistical analysis

To evaluate the case-based performance of the readers and the CAD, the accuracy, sensitivity, specificity, positive predictive value (PPV), and negative predictive value (NPV) were evaluated. A lung cancer patient with annotations with an IoU greater than or equal to 0.3 for a ground-truth lesion on a chest radiograph was defined as a true positive (TP) case, a lung cancer patient with annotations with an IoU less than 0.3 for a ground-truth lesion on a chest radiograph was defined as a false negative (FN) case, a non-lung cancer case with no annotations on a chest radiograph was defined as a true negative (TN) case, and a non-lung cancer case with one or more annotations on the chest radiograph was defined as a false positive (FP) case.

To evaluate the lesion-based performance of the readers and the CAD, we also determined the mean false positive indications per image (mFPI). The mFPI was defined as the value of the total false positive (FP) lesions divided by the total number of images. Annotated lesions were defined as FP if they had an IoU less than 0.3 with a ground-truth lesion. All annotations on a chest radiograph without lung cancer were defined as FP lesions.

These definitions are visually represented in Additional_File_2. In order to assess the improvement of readers’ performance metrics for detection of lung nodules due to the CAD, we determined the metrics for cases with and without CAD using Generalized Estimating Equations [26–28]. For each prediction metric, the performance with the CAD was divided by the performance without the CAD to assess the improved ratio. The statistical inferences were performed with two-sided 5% significance level. Decisions of readers before and after referencing CAD output were counted to evaluate the CAD effect. Two of the authors (D.U. and D.K.) performed all analyses using R, version 3.6.0.

## Results

### Datasets

From July 2017 through June 2018, we consecutively collected 122 chest radiographs from lung cancer patients. Eight radiographs were excluded because they contained metastases, 44 radiographs were excluded because the nodules were more than 30 mm in size, and four radiographs were excluded because the lesion showing was not nodular. The 66 remaining radiographs were annotated by author radiologists and seven radiographs were subsequently excluded because they concluded that the nodule was not visible on the chest radiograph. Thus, 59 radiographs from 59 patients were used as the malignant set. From July 2017 through June 2018, we collected 253 chest radiographs from patients with no nodule/mass finding via CT within 14 days. A total of 312 radiographs (59 malignant radiographs from 59 patients and 253 non-malignant radiographs from 253 patients; age range, 33–92 years; mean age ± standard deviation, 59 ± 13 years) were used for the test dataset to examine reader performance.

A flowchart of the eligibility criteria for the dataset is shown in Additional_File_3. Detailed demographic information of the test dataset is provided in Table 1.

**Table 1:** Dataset demographics

	Test dataset
Male	153
Female	159
Mean age ± standard deviation (years ± SD)	61.6 ± 11.4
Total no. of radiographs	312
Total no. of cancers	59
Size	
Average ± SD [mm]	17.9 ± 6.28
≦10 mm	7 (12%)
11–20 mm	33 (56%)
21–30 mm	19 (32%)
Laterality	
Right	39 (66%)
Left	20 (34%)
Location	
Upper	23 (39%)
Middle	31 (53%)
Lower	5 (8%)
Overlap	
Heart	2 (3%)
Clavicle	6 (10%)
Diaphragm	1 (2%)
Hilar vessels	3 (5%)
Manufacturer (% radiographs with cancer)	
FUJIFILM	6/89 (7%)
KONICA	31/113 (27%)
Philips	22/110 (20%)

Data are n unless otherwise noted. SD: standard deviation

### The deep learning-based computer-assisted detection model performance

The standalone CAD sensitivity, specificity, accuracy, PPV, and NPV were 0.66 (0.53–0.78), 0.96 (0.92–0.98), 0.90 (0.86–0.93), 0.78 (0.64–0.88), and 0.92 (0.88–0.95) with mFPI of 0.05, respectively.

### Reader performance test

**Fig. 1:**
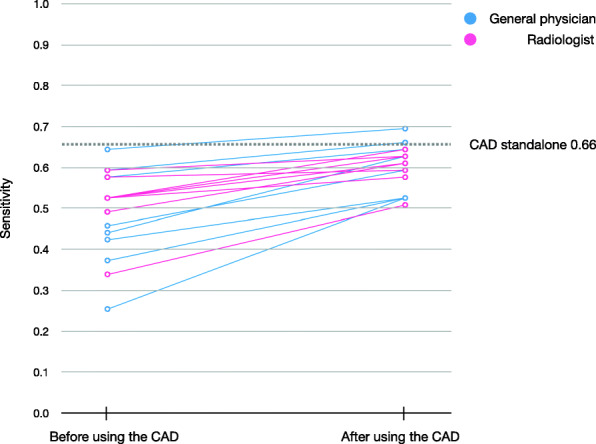
Sensitivity before and after using computer-assisted detection (CAD). The sensitivity to the test dataset before and after CAD use was plotted for each reader. Blue represents general physician and pink represents radiologist readers. For reference, the results of the CAD alone are shown by dotted lines

**Fig. 2:**
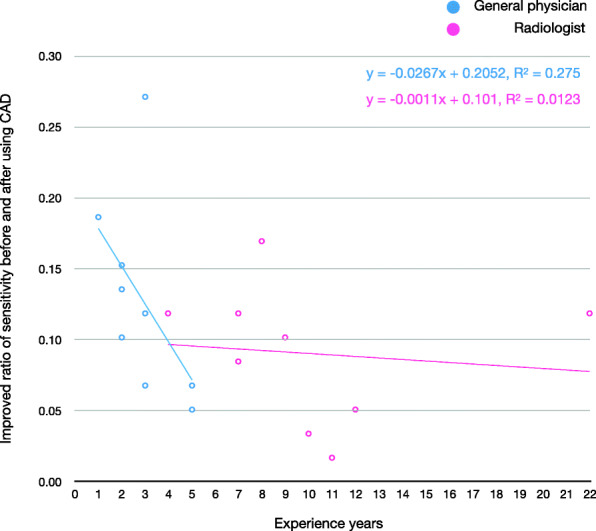
Improvement ratio for sensitivity and experience level of each reader. The rate of increase in sensitivity to the test dataset before and after computer-assisted detection (CAD) use was plotted for each reader. Blue represents general physician and pink represents radiologist readers. The trend lines for general physicians and radiologists are also shown

**Fig. 3:**
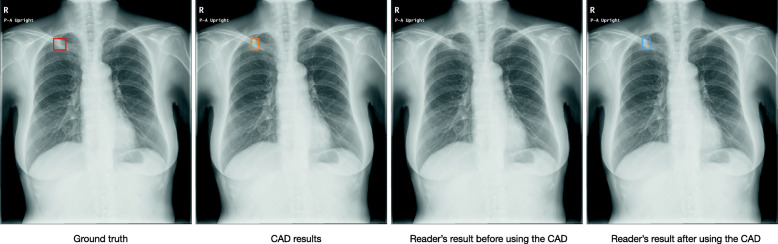
Example of a case in which physician correctly changed their decision due to computer-assisted detection (CAD) output. A case involving a 70-year-old woman with a nodule in the right upper pulmonary field overlapping the clavicle changed from false negative to true positive by a general physician with three years of experience (Reader 5), by referring to the true positive results of the CAD

The demographic information of the readers is provided in Supplementary Table 1 in Additional_File_1. All readers improved their overall performance by referring to the CAD output. The overall increases for reader performance due to using the CAD for sensitivity, specificity, accuracy, PPV, and NPV were 1.22 (1.14–1.30), 1.00 (1.00–1.01), 1.03 (1.02–1.04), 1.07 (1.03–1.11), and 1.02 (1.01–1.03), respectively (Table 2). General physicians benefited more from the use of the CAD than radiologists did. The performance of general physicians was improved from 0.47 to 0.60 for sensitivity, from 0.96 to 0.97 for specificity, from 0.87 to 0.90 for accuracy, from 0.75 to 0.82 for PPV, and from 0.89 to 0.91 for NPV while the performance of radiologists was improved from 0.51 to 0.60 for sensitivity, from 0.96 to 0.96 for specificity, from 0.87 to 0.90 for accuracy, from 0.76 to 0.80 for PPV, and from 0.89 to 0.91 for NPV. Detailed results per reader are in Supplementary Table 2 in Additional_File_1. The sensitivity of readers before and after using the CAD is shown as a bilinear graph in Fig. 1. The rate of improvement was particularly high for general physicians (Fig. 2). General physicians were more likely to change their assessment from FN to TP by referencing correct positive CAD output (68 times (0.59) in general physicians, 49 (0.49) in radiologists) and from FP to TN by correct negative CAD output (29 times (0.36) in general physicians, 24 times (0.29) in radiologists) (Table 3). The less experienced the reader was, the higher the rate of sensitivity improvement (Fig. 3). Conversely, the more experienced the readers were, the more limited the support capabilities of the CAD were. Radiologists were less likely to change their opinion than general physicians, and it was more difficult for radiologists to change their decisions from FP to TN (24 times) than from FN to TP (49 times). Results for readers’ determinations on TP radiographs were also calculated (Supplementary Table 3 in Additional_File_1). Additional_File_4 shows an instance in which a physician mistakenly changed their decision from TP to FN due to the FN output of the CAD. Instances in which physicians correctly changed their decision from FN to TP due to the TP output of the CAD can be seen in Fig. 3 and Additional_File_5.

**Table 2:** Results of readers with and without CAD

	General physicians		Radiologists		Overall		Ratio	95% Confidence Interval		
	Non-CAD	CAD	Non-CAD	CAD	Non-CAD	CAD	(CAD/Non-CAD)	Lower	Upper	*P* value
Sensitivity	0.47	0.60	0.51	0.60	0.49	0.60	1.22	1.14	1.30	< 0.001
Specificity	0.96	0.97	0.96	0.96	0.96	0.97	1.00	1.00	1.01	0.221
Accuracy	0.87	0.90	0.87	0.90	0.87	0.90	1.03	1.02	1.04	< 0.001
Positive Predictive Value	0.75	0.82	0.76	0.80	0.75	0.81	1.07	1.03	1.11	0.002
Negative Predictive Value	0.89	0.91	0.89	0.91	0.89	0.91	1.02	1.01	1.03	< 0.001

CAD: computer-assisted detection

**Table 3:** Decisions in readers before and after referencing CAD output

CAD results	TP		TN		FN		FP	
Reader results								
Before CAD use	FN		FP		TP		TN	
After CAD use	TP	FN	TN	FP	FN	TP	FP	TN
General physicians								
Reader 1	11	3	2	5	0	1	3	8
Reader 2	8	6	5	9	0	2	1	10
Reader 3	9	9	0	2	0	1	0	11
Reader 4	6	11	1	9	0	3	0	11
Reader 5	7	4	4	0	0	1	2	9
Reader 6	16	8	13	3	0	0	6	4
Reader 7	4	2	0	18	0	2	0	9
Reader 8	3	0	3	1	0	2	1	9
Reader 9	4	5	1	5	0	4	1	10
All general physicians	68	48	29	52	0	16	14	81
Radiologists								
Reader 10	7	4	2	13	0	1	4	7
Reader 11	5	4	0	6	0	1	0	11
Reader 12	7	5	2	0	0	2	2	9
Reader 13	11	10	11	2	1	1	2	9
Reader 14	6	4	0	13	0	2	7	3
Reader 15	2	6	5	7	0	4	2	8
Reader 16	1	8	0	2	0	4	0	11
Reader 17	3	8	0	10	0	3	0	11
Reader 18	7	3	4	5	0	2	4	5
All radiologists	49	52	24	58	1	20	21	74

Data are n. CAD: computer-assisted detection, FN: false negative, TP: true positive, FP: false positive, TN: true negative

## Discussion

We performed a multi-vendor, retrospective clinical validation to compare the performance of readers before and after using an AI-based CAD. The number of TPs that could be detected in the test dataset was greater than that of any human readers alone. The results of the present study indicate that the AI-based CAD can improve physician performance. Additionally, general physicians benefited more from the use of the CAD than radiologists did.

This is the first study to evaluate the performance not only of radiologists but also general physicians in their evaluation of chest radiographs with AI-based CAD assistance. A chest radiograph is one of the most basic tests that every physician is expected to be able to interpret to some extent, yet detection of pulmonary nodules on chest radiographs is prone to errors. Previous studies have found that about 20% of lung cancers visible on chest radiographs were actually missed at the time of initial reading [6, 8]. Physicians are aware of the risks misreading can cause, such as patient harm or medicolegal action, thus, the task can be difficult and distressing for inexperienced or general physicians. For this reason, we asked less experienced physicians to participate in this study to measure how much their performance could be improved with CAD support. Our results show that using this model could support both general physicians and radiologists in the detection of lung nodules.

The CAD increased physicians’ sensitivity with statistical significance without increasing the number of false positives. This is due to the high sensitivity of the CAD. The standalone CAD performance included a sensitivity of 0.66 (0.53–0.78) with mFPI of 0.05. This was comparable to or better than all of the individual physicians’ performance in our study. Since most AI models are designed to prevent misses, the trade-off is generally an increase in the number of false positives. These false positives can lead to an increase in unnecessary testing [29, 30]. This study indicates that more lung cancers could be detected without the need for chest CT or biopsy after implementation of this model into a chest radiography viewer.

To compare our results to previous CAD studies, this CAD shows a considerably lower mFPI. Previous studies showed an mFPI of 0.9–3.9 [18, 19, 31–37], while ours was 0.05. There are two studies [18, 19] with particularly high sensitivity and low mFPI. Sim et al. [19] showed a CAD sensitivity of 0.67 and an mFPI of 0.2, but their dataset excluded nodules smaller than 10 mm. Nam et al. [18] showed a CAD sensitivity of 0.69–0.82 and mFPI of 0.02–0.34, but their datasets contained a high percentage of masses greater than 30 mm and the nodules were not pathologically proven to be malignant. One possible reason why the CAD used in our study achieved high sensitivity with low mFPI was that it was created with a segmentation-based deep learning model, unlike other studies. Segmentation, also known as pixel labelling, deals with pixel-by-pixel information, which allows us to extract lesions more finely than general classification and detection models. The datasets in the former studies do not resemble a typical screening cohort. The sensitivity of the CAD in this study was found to be 0.66 with 0.05 mFPI. Although CAD has been applied to many fields, the typical increase in false positives remains a problem. This model was able to increase the sensitivity for true malignancies while reducing the number of false positives presented.

The advantage of using the AI model to the general physician was higher than that to the radiologist. In cases where the reader made a mistake (FN or FP) and the CAD showed the correct output (TP or TN), the general physicians were more likely to correct their error than the radiologists. Additionally, radiologists changed TN to FP more often (21 cases, or 22%) than general physicians (14 cases, or 15%) when the CAD presented FP output. The results showed that general physicians benefit more from this CAD than radiologists.

The limitations of this study include that the test dataset was collected from a single institution, although the readers who participated were from multiple institutions. The weakness of the CAD in detecting nodules of less than 10 mm may also be a limiting factor. The CAD could identify only one of the seven nodules under 10 mm, while most readers did not identify even one nodule. If the performance of CAD is improved, there is a possibility of detecting lung cancer at an earlier stage. Our dataset did not have radiographs with multiple lesions. In actual screening, single lesions are most common, but multiple lesions may be present.

## Conclusions

We conducted a multi-vendor, retrospective clinical validation to compare the performance of readers before and after using a commercially available AI-based CAD. The AI-based CAD supported physicians in the detection of lung cancers in chest radiography. We hope that the correct use of CAD in chest radiography, a basic and ubiquitous clinical examination, will lead to better medical care by preventing false negative assessments and supporting physicians’ determinations.

## Supplementary Information

**Additional File 1.** Supplementary Methods, Comments, Tables, and Supplementary Figure Legends

**Additional File 2.** Supplementary Fig. 1. Metric definitions for cases and lesions

**Additional File 3.** Supplementary Fig. 2. Eligibility of chest radiographs for test dataset

**Additional File 4.** Supplementary Fig. 3. Example of a case in which a physician mistakenly changed their decision from true positive to false negative due to the false negative output of the CAD

**Additional File 5.** Supplementary Fig. 4. Other examples of cases in which physicians correctly changed their decision from false negative to true positive due to the true positive output of the CAD

## Data Availability

The datasets used and/or analysed during the current study are available from the corresponding author on reasonable request. The commercial software used in this study is available from LPIXEL at https://eirl.ai/eirl-chest_nodule.

## Abbreviations

**AI**: Artificial intelligence
**CAD**: Computer-assisted detection
**DL**: Deep learning
**FN**: False negative
**FP**: False positive
**IoU**: Intersection over union
**mFPI**: Mean false positive indications per image
**NPV**: Negative predictive value
**PPV**: Positive predictive value
**TN**: True negative
**TP**: True positive
