# Supplementary material for: Artificial intelligence-supported lung cancer detection by multi-institutional readers with multi-vendor chest radiographs: a retrospective clinical validation study
Source: BMC Cancer. 2021 Oct 18;21:1120. doi: 10.1186/s12885-021-08847-9 (PMC8524996; doi:10.1186/s12885-021-08847-9)
Supplement: Supplementary file 1 — Additional File 1. Supplementary Methods, Comments, Tables, and Supplementary Figure Legends [file 12885_2021_8847_MOESM1_ESM.docx]

**TITLE**

Artificial intelligence-supported lung cancer detection by multi-institutional readers with multi-vendor chest radiographs: a retrospective clinical validation study

**TABLE OF CONTENTS**

**Section S1: Supplementary Methods**

**Section S2: Supplementary Comments**

**Section S3: Supplementary Tables 1–3**

Supplementary Table 1: Reader characteristics

Supplementary Table 2: Detailed results of readers with and without CAD

Supplementary Table 3: Detailed results of true positives

**Section S4: Supplementary Figures 1–4**

Supplementary Figure 1 in Additional_File_2: Metric definitions for case and lesion

Supplementary Figure 2 in Additional_File_3: Eligibility of chest radiographs for test dataset

Supplementary Figure 3 in Additional_File_4: Example of a case in which a physician mistakenly changed their decision from true positive to false negative due to the false negative output of the CAD

Supplementary Figure 4 in Additional_File_5: Other examples of cases in which physicians correctly changed their decision from false negative to true positive due to the true positive output of the CAD

**Supplementary Methods**

**Calculation of the number of cases**

In this study, the change in sensitivity was defined as the primary outcome. The required number of cases was set at a confidence level of 95%, based on the normal approximation method of binomial probability. Prior to this study, a preliminary performance test was conducted. Based on the results of the test, we set the expected sensitivity to 0.70 and specificity to about 0.90 with an estimated accuracy of 0.1.

Chest radiographs were consecutively collected of patients pathologically diagnosed with lung cancer within the inclusion period. Normal cases were also selected consecutively in the order of image acquisition until we reached the estimated number of cases required for statistical power.

If the number of images of lung cancer cases is too small, the power of the study may decrease due to the decreased accuracy of the sensitivity estimation. On the other hand, too many cases would increase the burden on reading physicians and may reduce his or her ability due to fatigue. Therefore, the number of cases considered appropriate was about 300.

Considering the above and the eligibility criteria mentioned in the main text, the final number of eligible images collected was 312 (253 normal cases and 59 lung cancer cases).

**Threshold of overlap with the ground truth**

We based our thresholds on the previous literature so we could compare our results to these studies. One of the two deep learning papers shown in the discussion used a threshold of 5% overlap with the ground truth [[1](https://pubs.rsna.org/doi/pdf/10.1148/radiol.2019182465)] and the other study used an IoU of 0.3 [[2](https://pubs.rsna.org/doi/pdf/10.1148/radiol.2018180237)].

One of the most famous studies in the medical AI field adopted an IoU of 0.1 [[3](https://www.nature.com/articles/s41586-019-1799-6)]. And they commented as follows:

“Similar work in three-dimensional chest computed tomography used any pixel overlap to qualify for correct localization. Likewise, an FDA-approved software device for the detection of wrist fractures reports statistics in which true positives require at least one pixel of overlap. An IoU value of 0.1 is strict by these standards.

**Supplementary Comments**

This study is different from that initially reported in 2019 (<https://eirl.ai/eirl-chest_nodule>). LPixel may choose to use our results after they are published.

**Supplementary References**

1. Sim Y, Chung MJ, Kotter E, Yune S, Kim M, Do S, et al. Deep convolutional neural network-based software improves radiologist detection of malignant lung nodules on chest radiographs. Radiology 2020;294:199–209.

2. Nam JG, Park S, Hwang EJ, Lee JH, Jin K, Lim KY, et al. Development and validation of deep learning-based automatic detection algorithm for malignant pulmonary nodules on chest radiographs. Radiology 2019;290:218–28.

3. McKinney SM, Sieniek M, Godbole V, Godwin J, Antropova N, Ashrafian H. et al. International evaluation of an AI system for breast cancer screening. Nature 2020;577:89–94.

**Supplementary Tables**

Supplementary Table 1. Reader characteristics

|  | Experience of physicians | Institution |
| --- | --- | --- |
| General physicians |  |  |
| Reader 1 | 1 years | A |
| Reader 2 | 2 years | D |
| Reader 3 | 2 years | F |
| Reader 4 | 2 years | B |
| Reader 5 | 3 years | B |
| Reader 6 | 3 years | C |
| Reader 7 | 3 years | G |
| Reader 8 | 5 years | A |
| Reader 9 | 5 years | G |
| Radiologists |  |  |
| Reader 10 | 4 years | I |
| Reader 11 | 7 years | I |
| Reader 12 | 7 years | E |
| Reader 13 | 8 years | A |
| Reader 14 | 9 years | I |
| Reader 15 | 10 years | I |
| Reader 16 | 11 years | H |
| Reader 17 | 12 years | A |
| Reader 18 | 22 years | A |

Supplementary Table 2. Detailed results of readers with and without CAD

|  |  | Sensitivity | Specificity | Accuracy | PPV | NPV | Mean FPI |
| --- | --- | --- | --- | --- | --- | --- | --- |
| CAD stand alone |  | 0.66 (0.53-0.78) | 0.96 (0.92-0.98) | 0.90 (0.86-0.93) | 0.78 (0.64-0.88) | 0.92 (0.88-0.95) | 0.05 |
| General physicians |  |  |  |  |  |  |  |
| Reader 1 | without CAD | 0.44 (0.31-0.58) | 0.97 (0.94-0.99) | 0.87 (0.83-0.91) | 0.79 (0.61-0.91) | 0.44 (0.31-0.58) | 0.03 |
|  | with CAD | 0.63 (0.49-0.75) | 0.97 (0.94-0.99) | 0.90 (0.87-0.93) | 0.82 (0.68-0.92) | 0.92 (0.88-0.95) | 0.05 |
| Reader 2 | without CAD | 0.46 (0.33-0.59) | 0.94 (0.91-0.97) | 0.85 (0.81-0.89) | 0.66 (0.49-0.80) | 0.46 (0.33-0.59) | 0.06 |
|  | with CAD | 0.59 (0.46-0.72) | 0.96 (0.93-0.98) | 0.89 (0.85-0.92) | 0.78 (0.63-0.89) | 0.91 (0.87-0.94) | 0.04 |
| Reader 3 | without CAD | 0.37 (0.25-0.51) | 0.99 (0.97-1.00) | 0.88 (0.83-0.91) | 0.92 (0.73-0.99) | 0.37 (0.25-0.51) | 0.01 |
|  | with CAD | 0.53 (0.39-0.66) | 0.99 (0.97-1.00) | 0.90 (0.87-0.93) | 0.94 (0.80-0.99) | 0.90 (0.86-0.93) | 0.01 |
| Reader 4 | without CAD | 0.42 (0.30-0.56) | 0.96 (0.93-0.98) | 0.86 (0.82-0.90) | 0.71 (0.54-0.85) | 0.42 (0.30-0.56) | 0.04 |
|  | with CAD | 0.53 (0.39-0.66) | 0.96 (0.93-0.98) | 0.88 (0.84-0.92) | 0.78 (0.62-0.89) | 0.90 (0.85-0.93) | 0.04 |
| Reader 5 | without CAD | 0.49 (0.36-0.63) | 0.98 (0.96-1.00) | 0.89 (0.85-0.92) | 0.88 (0.72-0.97) | 0.49 (0.36-0.63) | 0.01 |
|  | with CAD | 0.61 (0.47-0.73) | 0.99 (0.97-1.00) | 0.92 (0.88-0.95) | 0.95 (0.82-0.99) | 0.92 (0.88-0.95) | 0.02 |
| Reader 6 | without CAD | 0.25 (0.15-0.38) | 0.93 (0.89-0.96) | 0.80 (0.76-0.85) | 0.47 (0.29-0.65) | 0.25 (0.15-0.38) | 0.08 |
|  | with CAD | 0.53 (0.39-0.66) | 0.96 (0.93-0.98) | 0.88 (0.84-0.91) | 0.76 (0.60-0.88) | 0.90 (0.85-0.93) | 0.04 |
| Reader 7 | without CAD | 0.59 (0.46-0.72) | 0.92 (0.88-0.95) | 0.86 (0.82-0.90) | 0.64 (0.50-0.76) | 0.59 (0.46-0.72) | 0.07 |
|  | with CAD | 0.66 (0.53-0.78) | 0.92 (0.88-0.95) | 0.87 (0.83-0.91) | 0.66 (0.53-0.78) | 0.92 (0.88-0.95) | 0.08 |
| Reader 8 | without CAD | 0.64 (0.51-0.76) | 0.98 (0.95-0.99) | 0.92 (0.88-0.94) | 0.88 (0.75-0.96) | 0.64 (0.51-0.76) | 0.02 |
|  | with CAD | 0.69 (0.56-0.81) | 0.99 (0.97-1.00) | 0.93 (0.90-0.96) | 0.93 (0.81-0.99) | 0.93 (0.90-0.96) | 0.01 |
| Reader 9 | without CAD | 0.58 (0.44-0.70) | 0.98 (0.95-0.99) | 0.90 (0.86-0.93) | 0.85 (0.70-0.94) | 0.58 (0.44-0.70) | 0.03 |
|  | with CAD | 0.64 (0.51-0.76) | 0.98 (0.95-0.99) | 0.91 (0.88-0.94) | 0.86 (0.73-0.95) | 0.92 (0.88-0.95) | 0.04 |
| Radiologists |  |  |  |  |  |  |  |
| Reader 10 | without CAD | 0.49 (0.36-0.63) | 0.94 (0.90-0.97) | 0.86 (0.81-0.89) | 0.66 (0.50-0.80) | 0.49 (0.36-0.63) | 0.06 |
|  | with CAD | 0.61 (0.47-0.73) | 0.93 (0.89-0.96) | 0.87 (0.83-0.91) | 0.68 (0.54-0.80) | 0.91 (0.87-0.94) | 0.06 |
| Reader 11 | without CAD | 0.53 (0.39-0.66) | 0.98 (0.95-0.99) | 0.89 (0.85-0.92) | 0.84 (0.68-0.94) | 0.53 (0.39-0.66) | 0.02 |
|  | with CAD | 0.61 (0.47-0.73) | 0.98 (0.95-0.99) | 0.91 (0.87-0.94) | 0.86 (0.71-0.95) | 0.91 (0.87-0.95) | 0.02 |
| Reader 12 | without CAD | 0.49 (0.36-0.63) | 0.99 (0.97-1.00) | 0.90 (0.86-0.93) | 0.94 (0.79-0.99) | 0.49 (0.36-0.63) | 0.01 |
|  | with CAD | 0.61 (0.47-0.73) | 0.99 (0.97-1.00) | 0.92 (0.88-0.95) | 0.95 (0.82-0.99) | 0.92 (0.88-0.95) | 0.02 |
| Reader 13 | without CAD | 0.34 (0.22-0.47) | 0.95 (0.91-0.97) | 0.83 (0.79-0.87) | 0.61 (0.42-0.77) | 0.34 (0.22-0.47) | 0.08 |
|  | with CAD | 0.51 (0.37-0.64) | 0.98 (0.96-1.00) | 0.89 (0.85-0.93) | 0.88 (0.73-0.97) | 0.90 (0.85-0.93) | 0.02 |
| Reader 14 | without CAD | 0.53 (0.39-0.66) | 0.94 (0.91-0.97) | 0.87 (0.82-0.90) | 0.69 (0.53-0.82) | 0.53 (0.39-0.66) | 0.06 |
|  | with CAD | 0.63 (0.49-0.75) | 0.92 (0.88-0.95) | 0.86 (0.82-0.90) | 0.64 (0.50-0.76) | 0.91 (0.87-0.94) | 0.1 |
| Reader 15 | without CAD | 0.59 (0.46-0.72) | 0.95 (0.91-0.97) | 0.88 (0.84-0.92) | 0.73 (0.58-0.85) | 0.59 (0.46-0.72) | 0.06 |
|  | with CAD | 0.63 (0.49-0.75) | 0.96 (0.93-0.98) | 0.90 (0.86-0.93) | 0.79 (0.64-0.89) | 0.92 (0.88-0.95) | 0.05 |
| Reader 16 | without CAD | 0.58 (0.44-0.70) | 0.99 (0.97-1.00) | 0.91 (0.88-0.94) | 0.94 (0.81-0.99) | 0.58 (0.44-0.70) | 0.01 |
|  | with CAD | 0.59 (0.46-0.72) | 0.99 (0.97-1.00) | 0.92 (0.88-0.94) | 0.95 (0.82-0.99) | 0.91 (0.87-0.94) | 0.01 |
| Reader 17 | without CAD | 0.53 (0.39-0.66) | 0.96 (0.93-0.98) | 0.88 (0.84-0.91) | 0.76 (0.60-0.88) | 0.53 (0.39-0.66) | 0.04 |
|  | with CAD | 0.58 (0.44-0.70) | 0.96 (0.93-0.98) | 0.89 (0.85-0.92) | 0.77 (0.62-0.89) | 0.91 (0.87-0.94) | 0.04 |
| Reader 18 | without CAD | 0.53 (0.39-0.66) | 0.96 (0.92-0.98) | 0.88 (0.83-0.91) | 0.74 (0.58-0.86) | 0.53 (0.39-0.66) | 0.06 |
|  | with CAD | 0.64 (0.51-0.76) | 0.96 (0.93-0.98) | 0.90 (0.86-0.93) | 0.79 (0.65-0.90) | 0.92 (0.88-0.95) | 0.06 |

CAD: computer-assisted detection, PPV: positive predictive value, NPV: negative predictive value

Supplementary Table 3. Detailed results of true positives

|  |  | Size [mm] |  |  | Location |  |  | Overlap |  |  |  |  |  |  | Manufacture |  |  |
| --- | --- | --- | --- | --- | --- | --- | --- | --- | --- | --- | --- | --- | --- | --- | --- | --- | --- |
|  |  | 1-10 | 11-20 | 21-30 | Upper | Middle | Lower | Heart | Clavicle | Diaphragm | Nipple Diaphragm | Ribs | Hilar | No overlaps | FUJIFILM | KONICA | Philips |
| CAD standalone |  | 1/7 | 20/33 | 18/19 | 15/23 | 22/31 | 2/5 | 0/2 | 3/6 | 0/1 | 1/3 | 9/14 | 2/3 | 24/30 | 5/6 | 18/31 | 16/22 |
| General physicians |  |  |  |  |  |  |  |  |  |  |  |  |  |  |  |  |  |
| Reader 1 | without CAD | 0/7 | 15/33 | 11/19 | 10/23 | 14/31 | 2/5 | 0/2 | 1/6 | 0/1 | 1/3 | 6/14 | 2/3 | 16/30 | 3/6 | 11/31 | 12/22 |
|  | with CAD | 1/7 | 20/33 | 16/19 | 15/23 | 20/31 | 2/5 | 0/2 | 3/6 | 0/1 | 0/3 | 9/14 | 2/3 | 23/30 | 5/6 | 18/31 | 14/22 |
| Reader 2 | without CAD | 0/7 | 13/33 | 14/19 | 11/23 | 13/31 | 3/5 | 0/2 | 2/6 | 0/1 | 1/3 | 8/14 | 1/3 | 15/30 | 4/6 | 11/31 | 12/22 |
|  | with CAD | 0/7 | 19/33 | 16/19 | 13/23 | 19/31 | 3/5 | 0/2 | 2/6 | 0/1 | 1/3 | 8/14 | 2/3 | 22/30 | 5/6 | 16/31 | 14/22 |
| Reader 3 | without CAD | 0/7 | 10/33 | 12/19 | 8/23 | 12/31 | 2/5 | 0/2 | 1/6 | 0/1 | 1/3 | 6/14 | 1/3 | 13/30 | 3/6 | 8/31 | 11/22 |
|  | with CAD | 0/7 | 17/33 | 14/19 | 13/23 | 16/31 | 2/5 | 0/2 | 1/6 | 0/1 | 1/3 | 7/14 | 1/3 | 21/30 | 4/6 | 14/31 | 13/22 |
| Reader 4 | without CAD | 0/7 | 11/33 | 14/19 | 11/23 | 13/31 | 1/5 | 1/2 | 3/6 | 0/1 | 0/3 | 6/14 | 2/3 | 13/30 | 5/6 | 10/31 | 10/22 |
|  | with CAD | 0/7 | 16/33 | 15/19 | 12/23 | 18/31 | 1/5 | 1/2 | 3/6 | 0/1 | 0/3 | 7/14 | 2/3 | 18/30 | 5/6 | 16/31 | 10/22 |
| Reader 5 | without CAD | 0/7 | 14/33 | 15/19 | 11/23 | 17/31 | 1/5 | 0/2 | 2/6 | 0/1 | 1/3 | 5/14 | 2/3 | 19/30 | 4/6 | 13/31 | 12/22 |
|  | with CAD | 0/7 | 19/33 | 17/19 | 14/23 | 21/31 | 1/5 | 0/2 | 2/6 | 0/1 | 1/3 | 7/14 | 2/3 | 24/30 | 5/6 | 17/31 | 14/22 |
| Reader 6 | without CAD | 0/7 | 8/33 | 7/19 | 6/23 | 7/31 | 2/5 | 0/2 | 0/6 | 0/1 | 1/3 | 5/14 | 1/3 | 8/30 | 2/6 | 4/31 | 9/22 |
|  | with CAD | 0/7 | 18/33 | 13/19 | 12/23 | 17/31 | 2/5 | 0/2 | 1/6 | 0/1 | 1/3 | 7/14 | 2/3 | 20/30 | 5/6 | 14/31 | 12/22 |
| Reader 7 | without CAD | 2/7 | 17/33 | 16/19 | 12/23 | 21/31 | 2/5 | 0/2 | 2/6 | 0/1 | 1/3 | 9/14 | 2/3 | 21/30 | 5/6 | 14/31 | 16/22 |
|  | with CAD | 2/7 | 21/33 | 16/19 | 14/23 | 23/31 | 2/5 | 0/2 | 2/6 | 0/1 | 1/3 | 9/14 | 2/3 | 25/30 | 5/6 | 17/31 | 17/22 |
| Reader 8 | without CAD | 1/7 | 20/33 | 17/19 | 15/23 | 20/31 | 3/5 | 0/2 | 3/6 | 0/1 | 1/3 | 10/14 | 2/3 | 22/30 | 5/6 | 18/31 | 15/22 |
|  | with CAD | 1/7 | 22/33 | 18/19 | 15/23 | 23/31 | 3/5 | 0/2 | 3/6 | 0/1 | 1/3 | 10/14 | 2/3 | 25/30 | 5/6 | 19/31 | 17/22 |
| Reader 9 | without CAD | 0/7 | 19/33 | 15/19 | 12/23 | 19/31 | 3/5 | 0/2 | 3/6 | 0/1 | 1/3 | 8/14 | 2/3 | 20/30 | 4/6 | 15/31 | 15/22 |
|  | with CAD | 0/7 | 21/33 | 17/19 | 14/23 | 21/31 | 3/5 | 0/2 | 3/6 | 0/1 | 1/3 | 9/14 | 2/3 | 23/30 | 5/6 | 18/31 | 15/22 |
| Radiologists |  |  |  |  |  |  |  |  |  |  |  |  |  |  |  |  |  |
| Reader 10 | without CAD | 0/7 | 16/33 | 13/19 | 11/23 | 15/31 | 3/5 | 0/2 | 1/6 | 0/1 | 1/3 | 8/14 | 1/3 | 18/30 | 5/6 | 14/31 | 10/22 |
|  | with CAD | 0/7 | 19/33 | 17/19 | 15/23 | 18/31 | 3/5 | 0/2 | 3/6 | 0/1 | 1/3 | 8/14 | 1/3 | 23/30 | 5/6 | 17/31 | 14/22 |
| Reader 11 | without CAD | 0/7 | 15/33 | 16/19 | 11/23 | 18/31 | 2/5 | 0/2 | 2/6 | 0/1 | 1/3 | 6/14 | 2/3 | 20/30 | 5/6 | 13/31 | 13/22 |
|  | with CAD | 0/7 | 18/33 | 18/19 | 13/23 | 21/31 | 2/5 | 0/2 | 3/6 | 0/1 | 1/3 | 7/14 | 2/3 | 23/30 | 5/6 | 16/31 | 15/22 |
| Reader 12 | without CAD | 0/7 | 15/33 | 14/19 | 9/23 | 17/31 | 3/5 | 0/2 | 1/6 | 0/1 | 1/3 | 7/14 | 1/3 | 19/30 | 4/6 | 13/31 | 12/22 |
|  | with CAD | 0/7 | 20/33 | 16/19 | 13/23 | 20/31 | 3/5 | 0/2 | 2/6 | 0/1 | 1/3 | 8/14 | 2/3 | 23/30 | 5/6 | 16/31 | 15/22 |
| Reader 13 | without CAD | 0/7 | 9/33 | 11/19 | 7/23 | 11/31 | 2/5 | 0/2 | 2/6 | 0/1 | 1/3 | 5/14 | 2/3 | 10/30 | 2/6 | 8/31 | 10/22 |
|  | with CAD | 0/7 | 15/33 | 15/19 | 12/23 | 16/31 | 2/5 | 0/2 | 2/6 | 0/1 | 1/3 | 7/14 | 2/3 | 18/30 | 4/6 | 12/31 | 14/22 |
| Reader 14 | without CAD | 0/7 | 17/33 | 14/19 | 11/23 | 17/31 | 3/5 | 0/2 | 1/6 | 0/1 | 1/3 | 8/14 | 2/3 | 19/30 | 4/6 | 13/31 | 14/22 |
|  | with CAD | 0/7 | 21/33 | 16/19 | 14/23 | 20/31 | 3/5 | 0/2 | 2/6 | 0/1 | 1/3 | 8/14 | 2/3 | 24/30 | 5/6 | 17/31 | 15/22 |
| Reader 15 | without CAD | 0/7 | 20/33 | 15/19 | 15/23 | 18/31 | 2/5 | 0/2 | 3/6 | 0/1 | 0/3 | 9/14 | 2/3 | 21/30 | 5/6 | 16/31 | 14/22 |
|  | with CAD | 0/7 | 21/33 | 16/19 | 15/23 | 20/31 | 2/5 | 0/2 | 3/6 | 0/1 | 0/3 | 9/14 | 2/3 | 23/30 | 5/6 | 17/31 | 15/22 |
| Reader 16 | without CAD | 0/7 | 18/33 | 16/19 | 14/23 | 17/31 | 3/5 | 0/2 | 3/6 | 0/1 | 1/3 | 8/14 | 2/3 | 20/30 | 5/6 | 15/31 | 14/22 |
|  | with CAD | 0/7 | 19/33 | 16/19 | 14/23 | 18/31 | 3/5 | 0/2 | 3/6 | 0/1 | 1/3 | 8/14 | 2/3 | 21/30 | 5/6 | 16/31 | 14/22 |
| Reader 17 | without CAD | 0/7 | 18/33 | 13/19 | 11/23 | 17/31 | 3/5 | 0/2 | 2/6 | 0/1 | 1/3 | 8/14 | 2/3 | 18/30 | 5/6 | 12/31 | 14/22 |
|  | with CAD | 0/7 | 19/33 | 15/19 | 13/23 | 18/31 | 3/5 | 0/2 | 2/6 | 0/1 | 1/3 | 8/14 | 2/3 | 21/30 | 5/6 | 15/31 | 14/22 |
| Reader 18 | without CAD | 0/7 | 16/33 | 15/19 | 11/23 | 17/31 | 3/5 | 0/2 | 2/6 | 0/1 | 1/3 | 8/14 | 2/3 | 18/30 | 4/6 | 13/31 | 14/22 |
|  | with CAD | 1/7 | 21/33 | 16/19 | 14/23 | 21/31 | 3/5 | 0/2 | 2/6 | 0/1 | 1/3 | 9/14 | 2/3 | 24/30 | 5/6 | 17/31 | 16/22 |

CAD: computer-assisted detection

**Supplementary Figures**

**Supplementary Figure 1 in Additional_File_2. Metric definitions for case and lesion**

The gray rectangle represents the ground truth; the red rectangle represents the reader’s annotation. A true positive case has a ground truth with an overlapping annotation, while a true negative case has no ground truth nor annotation. A false negative case is a radiograph annotated with a ground truth that the reader did not identify correctly. A false positive case is a radiograph that the reader annotated, but no ground truth was marked.

**Supplementary Figure 2 in Additional_File_3. Eligibility of chest radiographs for test dataset**

Inclusion and exclusion criteria of chest radiographs.

**Supplementary Figure 3 in Additional_File_4. Example of a case in which a physician mistakenly changed their decision from true positive to false negative due to the false negative output of the CAD**

The red square indicates the location of the ground truth and the blue square indicates the location of the reader's interpretation.

Throughout this study, there was only one instance in which a physician mistakenly changed their decision from true positive to false negative due to the false negative output of the CAD. The reader is a radiologist with eight years of experience (Reader 13) and the case was a 60-year-old female with 16 mm nodule in the left lower lung field.

CAD: computer-assisted detection

**Supplementary Figure 4 in Additional_File_5. Other examples of cases in which physicians correctly changed their decision from false negative to true positive due to the true positive output of the CAD**

A red square indicates the location of the ground truth, a yellow square indicates the location of the CAD output result, and a blue square indicates the location of the reader's interpretation.

(a) A case involving a 71-year-old woman with a nodule in the left middle pulmonary field overlapping the hilar changed from false negative to true positive by a general physician with five years of experience (Reader 9), by referring to the true positive results of the CAD.

(b) A case involving a 73-year-old woman with a nodule in the left upper pulmonary field overlapping the ribs changed from false negative to true positive by a radiologist with four years of experience (Reader 10), by referring to the true positive results of the CAD.

CAD: computer-assisted detection
