# Supplementary material for: Artificial intelligence-supported lung cancer detection by multi-institutional readers with multi-vendor chest radiographs: a retrospective clinical validation study
Source: BMC Cancer. 2021 Oct 18;21:1120. doi: 10.1186/s12885-021-08847-9 (PMC8524996; doi:10.1186/s12885-021-08847-9)
Supplement: Supplementary file 2 — Additional File 2. Supplementary Fig. 1. Metric definitions for cases and lesions [file 12885_2021_8847_MOESM2_ESM.pdf]

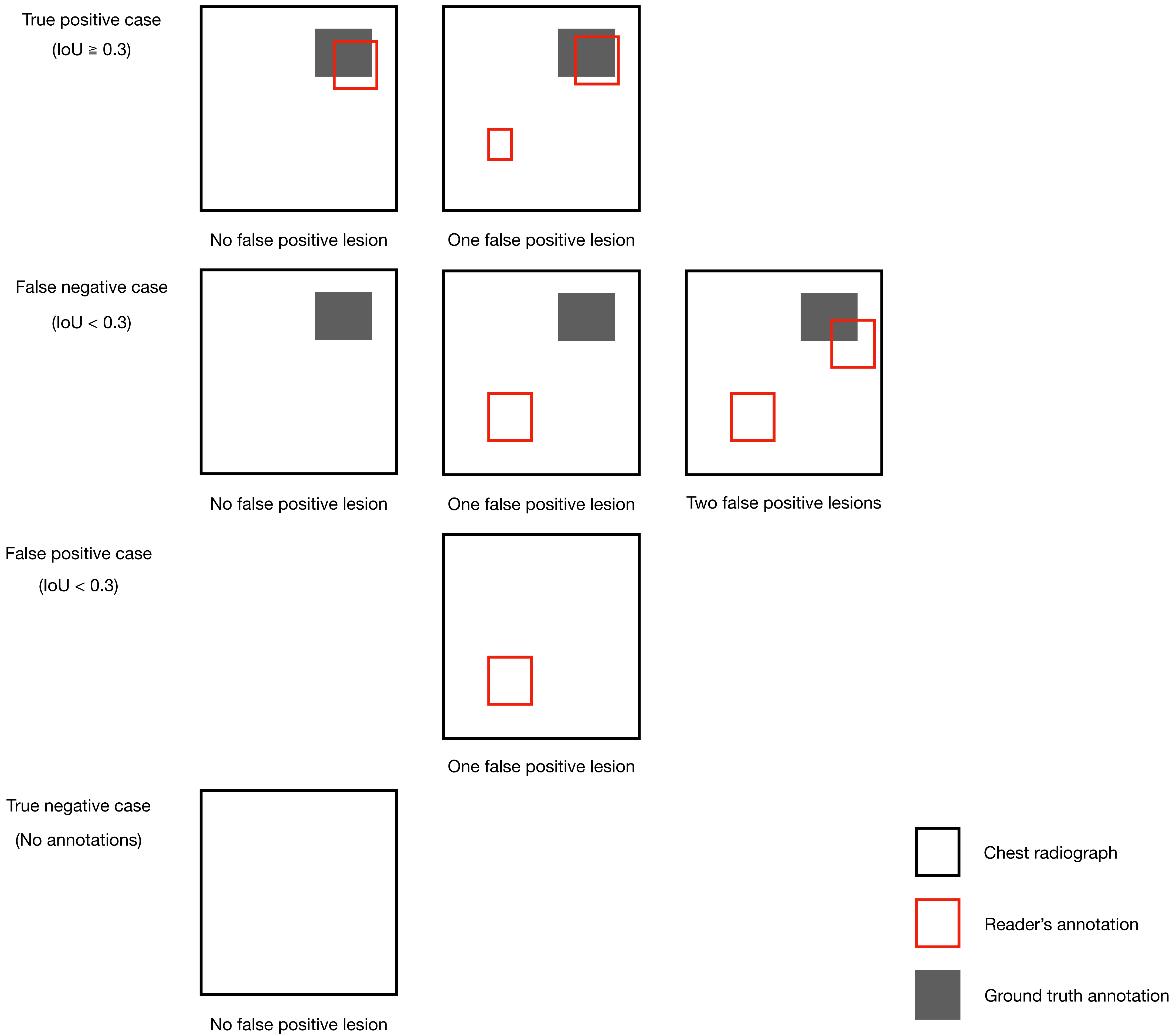
True positive case
(IoU ≧ 0.3)
No false positive lesion
One false positive lesion
False negative case
(IoU < 0.3)
No false positive lesion
One false positive lesion
Two false positive lesions
False positive case
(IoU < 0.3)
One false positive lesion
True negative case
(No annotations)
No false positive lesion
Chest radiograph
Reader's annotation
Ground truth annotation
