# Supplementary figures and images for: Artificial intelligence-supported lung cancer detection by multi-institutional readers with multi-vendor chest radiographs: a retrospective clinical validation study
Source: BMC Cancer. 2021 Oct 18;21:1120. doi: 10.1186/s12885-021-08847-9 (PMC8524996; doi:10.1186/s12885-021-08847-9)

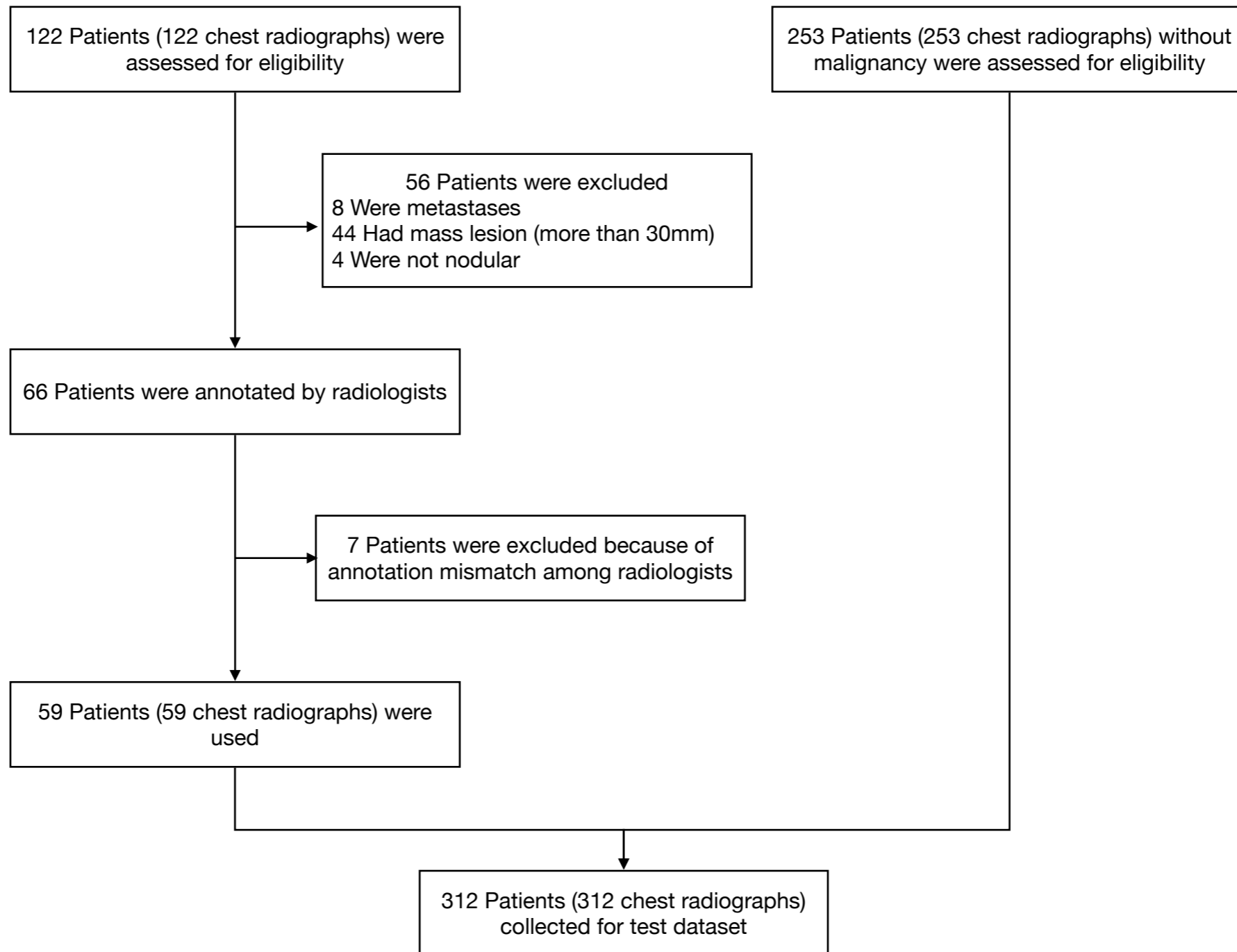

Supplement: Supplementary file 3 — Additional File 3. Supplementary Fig. 2. Eligibility of chest radiographs for test dataset [file 12885_2021_8847_MOESM3_ESM.pdf]
