# Supplementary material for: Artificial intelligence-supported lung cancer detection by multi-institutional readers with multi-vendor chest radiographs: a retrospective clinical validation study
Source: BMC Cancer. 2021 Oct 18;21:1120. doi: 10.1186/s12885-021-08847-9 (PMC8524996; doi:10.1186/s12885-021-08847-9)
Supplement: Supplementary file 4 — Additional File 4. Supplementary Fig. 3. Example of a case in which a physician mistakenly changed their decision from true positive to false negative due to the false negative output of the CAD [file 12885_2021_8847_MOESM4_ESM.pdf]

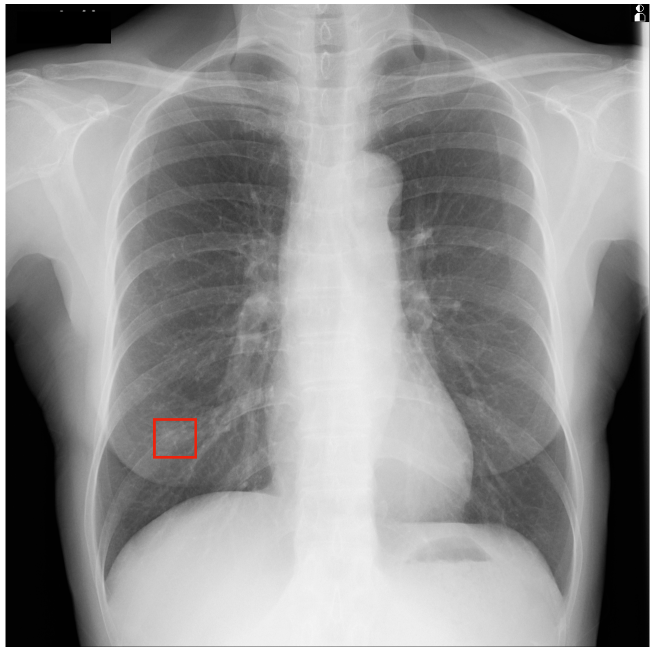

Ground truth

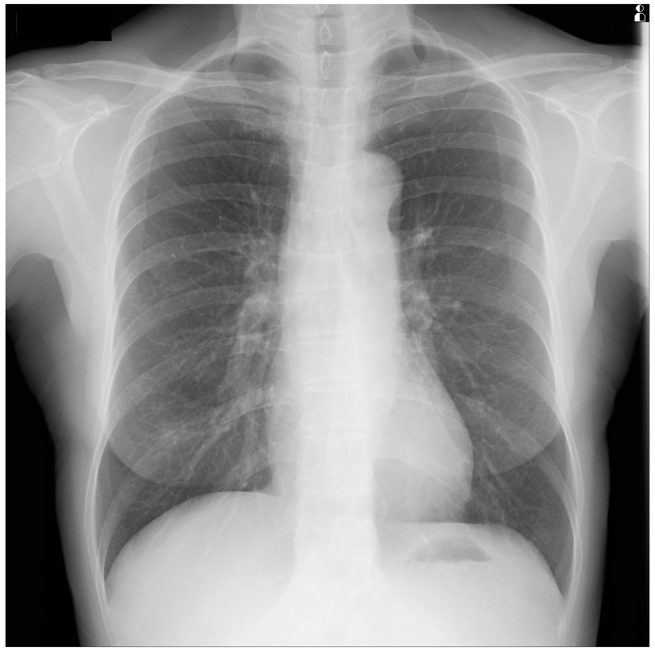

CAD results

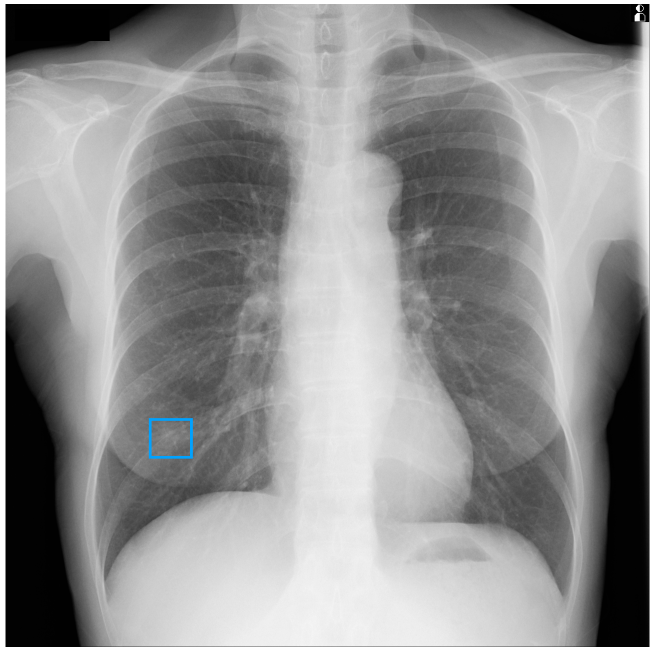

Reader’s result before using the CAD

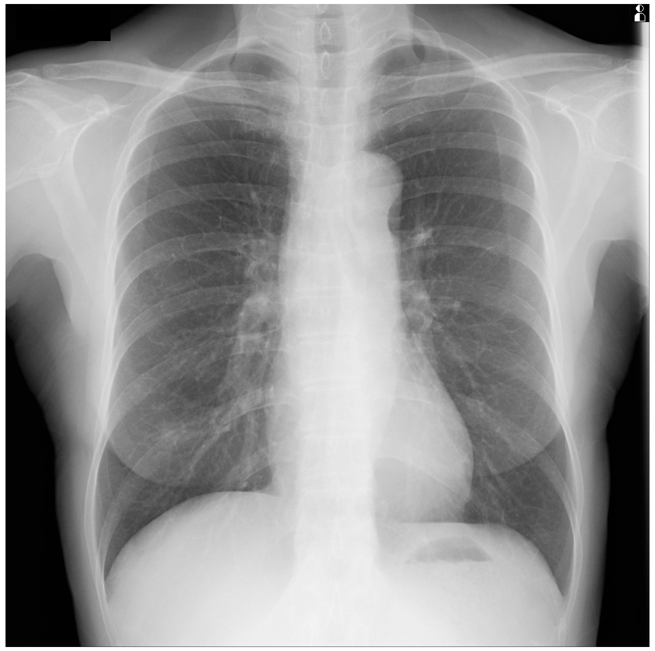

Reader’s result after using the CAD
