# Supplementary material for: Artificial intelligence-supported lung cancer detection by multi-institutional readers with multi-vendor chest radiographs: a retrospective clinical validation study
Source: BMC Cancer. 2021 Oct 18;21:1120. doi: 10.1186/s12885-021-08847-9 (PMC8524996; doi:10.1186/s12885-021-08847-9)
Supplement: Supplementary file 5 — Additional File 5. Supplementary Fig. 4. Other examples of cases in which physicians correctly changed their decision from false negative to true positive due to the true positive output of the CAD [file 12885_2021_8847_MOESM5_ESM.pdf]

(a)

(b)

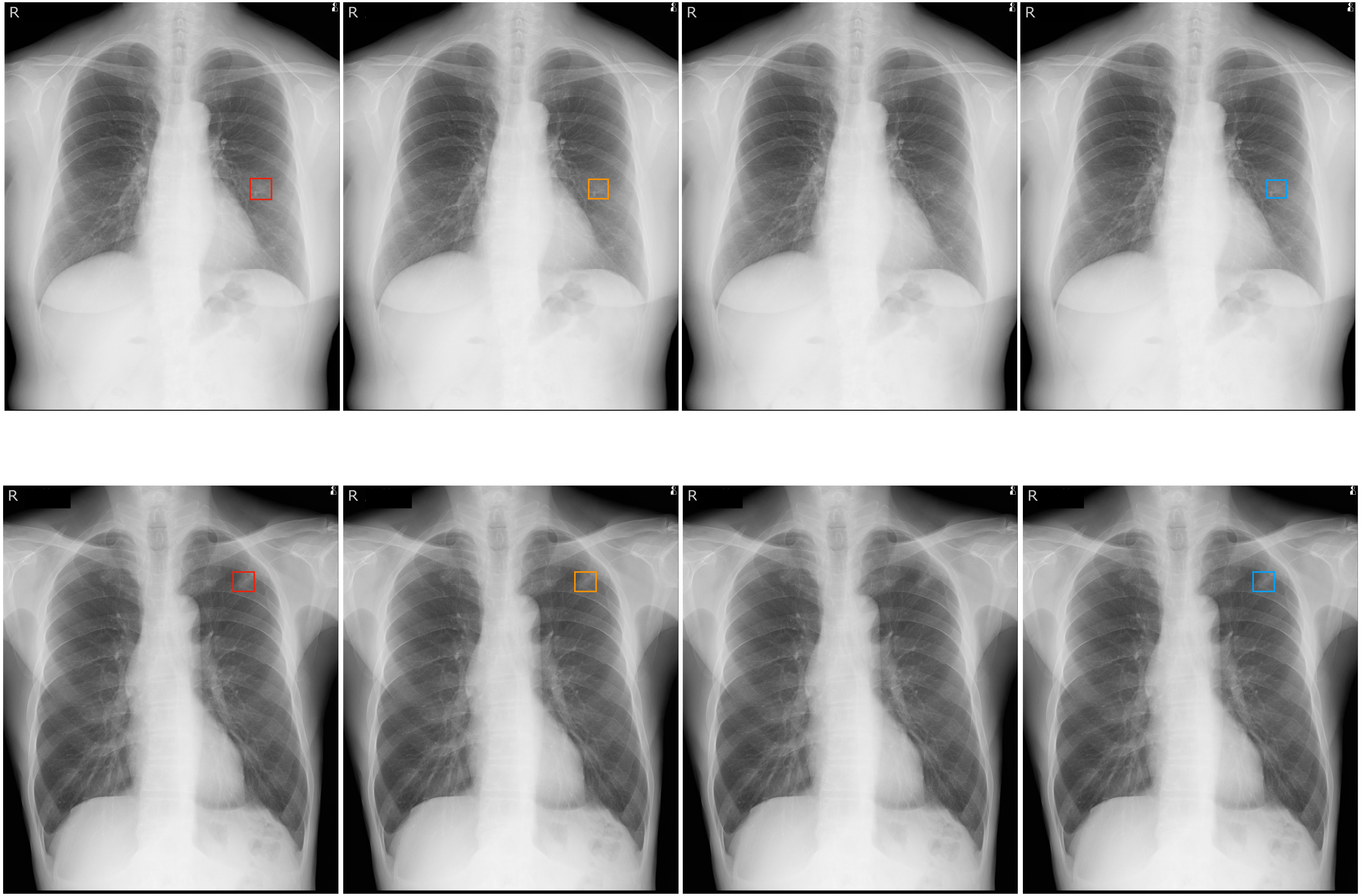

Ground truth

CAD results

Reader’s result before using the CAD

Reader’s result after using the CAD
